# Supplementary material for: WordCommentsAnalyzer: A windows software tool for qualitative research
Source: F1000Res. 2018 Sep 4;7:536. Originally published 2018 May 3. [Version 2] doi: 10.12688/f1000research.14819.2 (PMC6137411; doi:10.12688/f1000research.14819.2)
Supplement: Supplementary file 3 [file f1000research-7-17614-s0002.tgz › b2a79496-4ada-47c4-9682-a99ce867836d.zip!/Eight Iranian Health Journals/Yafteh _21.docx]

© Yafteh  . This work is licensed under CC BY 4.0 (https://creativecommons.org/licenses/by/4.0/).

Volume & Issue: دوره 15 - شماره 2

Title: بررسی میزان ویتامین‌ ث، اسیدهای آلی، ترکیبات فرار و معطر میوه سیب (Malus domestica Borkh) ارقام زرد و قرمز لبنانی در استان لرستان

http://yafte.lums.ac.ir/article-1-1261-fa.html

مقدمه: سیب مهمترین میوه مصرفی در تعداد زیادی از کشورهای جهان می باشد و اهمیت بسیار زیادی در رژیم غذایی و سلامت فرد دارد. این تحقیق با هدف تعیین میزان ویتامین ث (اسید اسکوربیک)، اسیدهای آلی غالب (اسید سیتریک و مالیک)، و ترکیبات فرار و معطر میوه سیب ارقام زرد و قرمز لبنانی در شهرستان‌های خرم آباد و بروجرد انجام گرفت. مواد و روش‌ها: در این مطالعه مقطعی نمونه‌های میوه مورد نیاز در اواخر شهریورماه سال 1387 جمع آوری شد. برای این منظور از هر درخت که به عنوان یک واحد آزمایشی محسوب شد و تعداد 5 عدد سیب در مرحله بلوغ فیزیولوژیکی چیده شد. ترکیبات فرار و معطر با استفاده از روش جدید ریز استخراج با فاز جامد از فضای فوقانی – مافوق صوت (SPME GC/MS)، میزان اسید آسکوربیک (ویتامین ث) با استفاده از روش 2 ،6- دی کلروفنل اندوفنل و میزان اسیدهای آلی میوه (اسید مالیک و سیتریک) به روش تیتراسیون اندازه‌گیری شد. یافته‌ها: مقدار اسید اسکوربیک در سیب قرمز و زرد به ترتیب 49/9 و 09/9 میلی گرم در 100 گرم و در مجموع 29/9 میلی گرم در 100 گرم بود. مقدار اسید مالیک در سیب قرمز و زرد به ترتیب 26/0 و 27/0 و اسید سیتریک در هر دو نوع سیب 28/0 میلی گرم در 100 گرم بود . اسیدیته سیب قرمزدر حدود 4 و سیب زرد 7/3 بود. سیب قرمز دارای اسیدیته بیشتری نسبت به سیب زرد بود. از نظر مواد فرار بیشترین فراوانی در بین سیب قرمز و زرد مربوط به فارنسین بود. بحث و نتیجه‌گیری: در مجموع سیب استان لرستان دارای اسید اسکوربیک در حدود سه برابر مقدار موجود در جدول ترکیبات مواد غذایی ایران است. از نظر اسید مالیک و سیتریک تفاوت معنی داری بین سیب زرد و قرمز وجود نداشت.

Title: استخراج وشناسایی ترکیبات مؤثر عصاره هیدروالکلی تاراکساکوم سیریاکوم (قاصدک) و اثر محافظتی آن بر مسمومیت کلیوی ناشی از استامینوفن در موش صحرایی نر

http://yafte.lums.ac.ir/article-1-1262-fa.html

مقدمه: گیاهBoiss Taraxacum syriacum از گونه Taraxacum officinalis (قاصدک) است که در طب سنتی برای درمان یرقان و اختلالات کبدی و سنگ صفرا مورد استفاده قرار می گیرد. در تحقیق حاضر اثر محافظتی عصاره هیدروالکلی ریشه گیاهBoiss Taraxacum syriacum (TSBE) در مسمومیت کلیوی ناشی از استامینوفن مورد بررسی قرار گرفت. بحث و نتیجه‌گیری: عصاره هیدروالکلی ریشه گیاه Taraxacum syriacum Boiss در مسمومیت کلیوی ناشی از استامینوفن در موشهای صحرایی تا حدودی نقش محافظتی دارد که به نظر می رسد در این میان نقش ترکیبات فنولی از جمله کارواکرول آن حائز اهمیت است. یافته‌ها: با استفاده از روش ریز استخراج بیست و یک ترکیب مختلف شیمیایی از جمله 1- 1 دی متیل دیبوران، 1 پروپن 3 اتوکسی، 3-5 اکتادین دوآن، نونانال، دکانال، نونانوئیک اسید و کارواکرول در نمونه ریشه گیاه استخراج و شناسایی گردید. کراتینین در TSBE با دوز دویست میلی گرم بر کیلوگرم نسبت به گروه مسموم کاهش معنی دار پیدا کرده بود. در مطالعات هیستوپاتولوژیک نیز، نکروز کلیوی در گروه‌های عصاره (100و200 میلی گرم بر کیلوگرم) کاهش یافته بود. مواد و روش‌ها: استخراج و شناسایی ترکیبات ریشه گیاه با استفاده از روش SPME-GC/MS (Solid Phase Microextraction ) انجام گرفت. در این تحقیق تجربی آزمایشگاهی 30 موش نر بالغ به طور تصادفی پنج گروه تقسیم شدند (6= n). حیوانات در ابتدا با مقادیر مختلف TSBE (50، 100 و 200 میلی گرم بر کیلوگرم، گاواژ) روزانه به مدت هفت روز پیاپی تیمار شدند، سپس در معرض دوز سمی استامینوفن (APAP) ( 700 میلی گرم بر کیلوگرم داخل صفاقی) قرار گرفتند. در گروه کنترل آب مقطر به مدت هفت روز تجویز شد. در گروه سمی استامینوفن آب مقطر به مدت هفت روز دریافت و در روز هفتم دوز سمی استامینوفن تزریق شد. بیست و چهار ساعت بعد نمونه خونی برای اندازه گیری کراتینین سرمی مجزا گردید و کلیه موش‌ها جدا شده و برای بررسی های هیستوپاتولوژیک در فرمالین ده درصد قرار داده شدند.

Title: اثرات مهاری ساچوریا خوزستانیکا (Satureja khozestanika) بر اکسیداسیون LDLسرم تحت اثر سولفات مس در محیط برون‌تنی

http://yafte.lums.ac.ir/article-1-1263-fa.html

مقدمه: اکسیداسیون لیپیدها و ازجمله LDL نقش مهمی در ایجاد آترواسکلروز دارد. با استفاده از مواد آنتی اکسیدان در جیره غذایی مانند ویتامین E و اسانس گیاهانی مانند اسانس مرزه خوزستانی می توان از اکسیداسیون LDL جلوگیری و در نتیجه باعث جلوگیری از ایجاد و پیشرفت آترواسکلروز شد. هدف از این تحقیق بررسی اثر غلظت های مختلف اسانس روغنی مرزه خوزستانی (ساچوریا خوزستانیکا) بر مهار بر اکسیداسیون LDL سرم درمحیط برون تنی است. بحث و نتیجه‌گیری: نتایج بدست آمده نشان میدهد که اسانس روغنی مرزه خوزستانی باعث جلوگیری ازاکسیداسیون LDL واین ترکیبات ممکن است اثرات مشابهی در شرایط درون تنی داشته باشد. یافته‌ها: نتایج بدست آمده نشان می دهد که اسانس روغنی مرزه خوزستانی باعث کاهش اکسیداسیون LDL سرم می شود، بطوری که میزان زمان تاخیری براساس غلظت اسانس روغنی مرزه خوزستانی (50، 100و 200 میکروگرم بر میلی لیترو ویتامین Eباغلظت 100میکرومولار) به ترتیب 33/33، 66/66، 100 و11/111 درصد افزایش یافت. اثر مهاری اسانس روغنی مرزه خوزستانی بر اکسیداسیون LDL متناسب با غلظت (50، 100و 200 میکروگرم برمیلی لیتر) است. مواد و روش‌ها: از افراد سالم بعد از یک شب ناشتایی نمونه خون تهیه شد و LDL سرم جداشده را در گروه های کنترل ، اکسید شده با مس و اسانس روغنی مرزه خوزستانی با غلظت های (50، 100 و 200 میکروگرم برمیلی لیتر) و ویتامین E با غلظت 100 میکرومولار به عنوان کنترل مثبت مطالعه شد. جهت بررسی اکسیداسیون LDL مقدار دی ان های کونژوگه، زمان تاخیری ((Lag time و MDA (مالون دی آلدئید) تشکیل شده اندازه گیری شد و اثرات غلظت های مختلف اسانس روغنی مرزه خوزستانی و ویتامین Eبر مهار اکسیداسیون LDL سرم تحت اثر مس بررسی شد.

Title: اسید گالیک حافظه و درد را در موش های صحرایی نر دیابتی بهبود می‌بخشد

http://yafte.lums.ac.ir/article-1-1264-fa.html

مقدمه: عوارض دیابت ممکن است ناشی از تولید رادیکال‌های آزاد باشد که سبب بروز مشکلات حافظه می‌شود و ابتلا به زوال عقل را افزایش ‌می‌دهد. هم چنین افراد دیابتی در معرض خطر دردهای عصبی هستند. اسیدگالیک دارای خواص آنتی‌اکسیدانی و ضد رادیکال‌های آزاد است. در این مطالعه اثر تجویز اسیدگالیک ، بر حافظه اجتنابی غیرفعال و درد در موش‌های دیابتی بررسی گردید. مواد و روش‌ها: حیوانات به گروه‌های کنترل (سالم)، دیابتی با STZ (mg/kg60)، 3 گروه سالم و 3 گروه دیابتی که به مدت دو هفته اسید-گالیک (mg/kg 10، 50 و100) را به روش گاواز دریافت کردند ، تقسیم شدند. میزان قند‌خون با خون‌گیری از ناحیه دم اندازه‌گیری ‌شد. بحث و نتیجه‌گیری: با توجه به یافته های این تحقیق اسیدگالیک احتمالاً با اثر آنتی‌اکسیدانی قوی منجر به جاروب رادیکال‌های آزاد و کاهش عوارض ناشی از دیابت از جمله درد می گردد و احتمالاً بر مسیرهای عصبی حافظه در نواحی خاص مغز اثر می کند و سبب بهبود حافظه در موش‌های سالم و دیابتی می شود. یافته‌ها: یافته‌‌ها کاهش معنی‌دار حافظه (تأخیر در پایین آمدن از سکو) در گروه دیابتی در تمامی روزها به جز روز یادگیری (P≤0.01) را نشان‌داد. دوز 50 اسیدگالیک افزایش معنی‌داری در حافظه موش‌های غیردیابتی در روز اول(P≤0.01)، سوم و هفتم(P≤0.05) و نیز دوز 10 در روز اول (P≤0.05) ایجاد‌نمود. هم چنین افزایش معنی‌دار حافظه‌ روز اول(P≤0.01)، سوم و هفتم(P≤0.05) گروه‌های دیابتی دریافت-کننده‌ دوز 50 و10 اسید گالیک مشاهده‌گردید. در رفلکس دورکشیدن دم( تاخیر در پرتاب دم) از کانون درد کاهش معنی‌داری(P≤0.01) در گروه دیابتی مشاهده شد و تنها در گروه دریافت کننده دوز 50 اسیدگالیک، افزایش معنی‌داری(P≤0.01) در تاخیر در پرتاب دم مشاهده گردید.

Title: فعالیت ضد باکتریایی عصاره‌ی آبی گال‌های قلقاف و مازوج درختان بلوط از جنگل‌های لرستان

http://yafte.lums.ac.ir/article-1-1265-fa.html

مقدمه: امروزه عفونت‌های باکتریایی و مقاومت آنتی بیوتیکی عوامل‌ایجاد کننده آن‌ها از مهم‌ترین چالش‌های پیش روی سیستم‌های بهداشتی می‌باشند. از‌ این رو اخیراً استفاده از عوامل ضد میکروبی با منشا طبیعی مورد توجه بسیاری قرار گرفته است. هدف از‌این تحقیق بررسی اثر ضد باکتریایی عصاره‌ی آبی گال‌های مازوج و قلقاف بر باکتری‌های گرم منفی بود. مواد و روش‌ها: فعالیت ضد باکتریایی عصاره آبی دو گال بر سویه‌های باکتریایی جدا شده از نمونه‌های بالینی با مقاومت دارویی چندگانه (Pseudomonas aeruginosa, Acinetobacter baumannii, Escherichia coli ) و سویه‌های باکتریایی مرجع به روش آگار دیفیوژن و آگار دایلوشن تعیین گردید. بحث و نتیجه‌گیری:‌این بررسی نشان داد که گال‌های مازوج و قلقاف منابعی از ترکیبات ضد باکتریایی می‌باشند که می‌توان از آن‌ها علیه باکتری‌های گرم منفی با مقاومت چند گانه استفاده نمود؛ لذا مطالعه بر روی فعالیت ضد باکتریایی سایر عصاره‌های‌ این دو گال و بررسی‌های فارماکولوژیک ضروری می‌نماید. یافته‌ها: عصاره آبی هر دو گال فعالیت ضد باکتریایی بالایی را علیه تمام سویه‌های مورد بررسی نشان دادند. قطر هاله عدم رشد برای عصاره آبی گال‌های قلقاف ومازوج به ترتیب در محدوده 20-8 و 26-8 میلی متر بود. اندازه‌ی قطر هاله‌ی عدم رشد با غلظت عصاره نسبت مستقیم داشت (05/0>p). پایین ترین میزان MIC مربوط به عصاره آبی گال مازوج (mg/ml 128/0) بود.

Title: بررسی فعالیت ضد باکتریایی عصاره هیدروالکلی خرزهره (Nerium oleander)

http://yafte.lums.ac.ir/article-1-1266-fa.html

مقدمه: در طب باستانی اعراب و چین از گیاه خرزهره برای درمان بیماری‌ها استفاده می‌شود. در این تحقیق خواص ضد باکتریایی عصاره هیدروالکلی خرزهره بومی ایلام بر روی باکتری‌های بیماری‌زا مورد بررسی قرار گرفت. بحث و نتیجه‌گیری: نتایج به دست آمده نشان می‌دهد که باکتری‌های گرم مثبت نسبت به عصاره خرزهره حساس‌تر از باکتری‌های گرم منفی می‌باشند. و با توجه به قطرهاله‌های عدم رشد باکتری‌ها می‌توان عنوان کرد که عصاره هیدروالکلی خرزهره اثر باکتری کشی قابل توجه بر باکتری‌های تحت مطالعه دارد. یافته‌ها: بیشترین تاثیر عصاره هیدروالکلی گیاه خرزهره در غلظت 76 میلی‌گرم بر میلی‌لیتر بود. بیشترین قطر هاله عدم رشد در غلظت 76 میلی‌گرم مربوط به باکتری 2321E. faecalis (05/0p<) و کمترین قطر در همین غلظت مربوط به P. aeruginosa بود. نتایج نشان داد که کمترین میزان MIC مربوط به باکتری 1885S. aureus در غلظت 5 میلی‌گرم بر میلی‌لیتر بوده و بیشترین میزان MIC مربوط به باکتری E. coli و P. aeruginosa در غلظت 76 میلی‌گرم می‌باشد (05/0p<). مواد و روش‌ها: گیاه مذکور از کوههای زاگرس شهر ایلام جمع آوری و پس از شناسایی و نامگذاری، عصاره به روش ماسراسیون تهیه شد. غلظت‌های مختلف عصاره با استفاده از روش دیسک دیفیوژن بر روی باکتری‌ها تاثیر داده شد. از آنتی بیوتیک‌های تشخیصی به عنوان کنترل مثبت و از DMSO به عنوان کنترل منفی استفاده گردید. میزانMIC و MBC نیز مشخص شد.

Title: بررسی اثرات آنتی باکتریال عصاره آبی یونجه Medicago sativa L.در مقایسه با سه آنتی‌بیوتیک جنتامایسین، سیپروفلوکسازین و پنی سیلین

http://yafte.lums.ac.ir/article-1-1267-fa.html

مقدمه: در سال های اخیر، استفاده از مواد طبیعی به جای داروهای شیمیایی سنتتیک دارای عوارض جانبی، در پیشگیری و کنترل عفونت های مختلف تاکید شده است. با افزایش روز افزون مصرف گیاهان دارویی در درمان طبی، این شاخه از طب مکمل، جایگاه ویژه‏ای در درمان بیماری‏ها پیدا کرده است. تحقیق حاضر به منظور بررسی اثر ضد میکروبی عصاره آبی بذر یونجه در مقایسه با آنتی بیوتیک های جنتامایسین، پنی سیلین و سیپروفلوکسازین بر روی سویه های استاندارد باکتری های سودوموناس آئروژینوزا، اشرشیا کلی، استافیلوکوکوس اورئوس، لیستریا مونوسیتوژنز صورت پذیرفت. مواد و روش‌ها: آزمایشات با استفاده از روش های دیسک دیفیوژن و میکروبراث دایلوشن انجام شد و MIC (حداقل غلظت مهارکننده رشد) و MBC (حداقل غلظت کشنده) گیاه شناسایی شدند. بحث و نتیجه‌گیری: بر اساس یافته های این تحقیق عصاره آبی گیاه مذکور می تواند کاندید مناسبی برای بررسی های آزمایشگاهی و تکمیلی جهت جداسازی ترکیبات فعال موجود در آن برای دستیابی به داروهای ﻣﺆثر ضد میکروبی باشد. یافته‌ها: حداقل غلظت مهارکننده رشد عصاره آبی گیاه یونجه بر باکتری های گرم مثبت لیستریا منوسایتوژنز و استافیلوکوکوس اورئوس به ترتیب 25/206 و6600 میکروگرم بر میلی بوده و بر باکتری های گرم منفی تست شده در این مطالعه تاثیری نداشت.

Title: مقایسه تأثیر گل‌مغربی، ویتاگنوس و ویتامین E بر افسردگی زنان: کارآزمایی بالینی

http://yafte.lums.ac.ir/article-1-1268-fa.html

مقدمه: شمار قابل توجهی از مردم، برای درمان بیماری‌های مختلف، در جستجوی جایگزینی برای طب رایج، می باشند. افسردگی یکی از بیماری‌هایی است که در زنان دو برابر مردان دیده می‌شود و یکی از علل آن، اختلالات هورمونی می‌باشند. از آنجا که گیاهان دارویی در درمان این اختلالات مؤثرند؛ این مطالعه با هدف بررسی تأثیر سه داروی ویتاگنوس، گل مغربی و ویتامین E بر افسردگی زنان مبتلا به درد سینه دوره‌ای انجام شد. بحث و نتیجه‌گیری: نتایج پژوهش، نشان داد که دو داروی گیاهی به میزان مشابه، باعث کاهش افسردگی شده‌اند. از آنجا که از نظر اقتصادی مصرف داروی ویتاگنوس بسیار با صرفه‌تر است؛ توصیه می‌شود از این دارو در زنان مبتلا به افسردگی استفاده شود. یافته‌ها: تفاوت معنی‌داری در سطح افسردگی بیماران، قبل از مداخله درمانی و بعد از آن، در دو گروه گل‌مغربی و ویتاگنوس مشاهده شد. در گروه ویتامین E، تفاوت آماری معنی‌داری قبل از مداخله درمانی و بعد از درمان، مشاهده نشد. مواد و روش‌ها: در این کارآزمایی بالینی، 210 زن مبتلا به ماستالژی دوره‌ای که دارای معیارهای ورود به مطالعه بودند؛ به روش نمونه‌گیری آسان انتخاب و به‌صورت تصادفی به سه گروه، تقسیم شدند. در ابتدا، سطح افسردگی به‌وسیله پرسشنامه بک سنجیده و سپس به مدت سه ماه، در گروه اول، داروی ویتاگنوس، در گروه دوم، کپسول روغن گل‌مغربی و در گروه سوم، ویتامین E داده شد. در پایان مجدداً امتیاز افسردگی، سنجیده و از طریق تست‌های آماری با هم مورد مقایسه قرار گرفت.

Title: فعالیت ضد باکتریایی عصاره‌متانولی و استونی گال خرنوک درخت بلوط علیه باکتری‌های گرم مثبت

http://yafte.lums.ac.ir/article-1-1269-fa.html

بحث و نتیجه­گیری : عصاره‌های متانولی و استونی گال‌خرنوک بر روی همه‌ باکتری‌های مورد آزمایش فعالیت ضد باکتریایی نشان دادند. بنابراین، نتایج حاصل از این تحقیق نشان می‌دهد که عصاره‌های متانولی و استونی گال خرنوک از بلوط Quercus infectoria را می توان به عنوان یک ترکیب ضد باکتریایی در درمان پیشنهاد نمود.   مواد و روش ها : این مطالعه تجربی آزمایشگاهی در پاییز 1390 انجام شد. گال­ها از جنگل‌های بلوط لرستان جمع‌آوری شد. گال‌، خشک و آسیاب گردید و با حلال‌های متانول و استون به روش خیساندن عصاره‌گیری شد. غلظت‌های 50، 25، 5/12، 25/6، 12/3، 56/1 میلی‌گرم بر میلی‌لیتر از عصاره‌های متانولی و استونی به روش سری رقت تهیه گردید. خاصیت ضد باکتریایی عصاره‌های حاصل در غلظت‌های مختلف، با استفاده از روش انتشار چاهک بر باکتری‌های مذکور، مورد بررسی قرار گرفت. روش میکرودایلوشن برای تعیین حداقل غلظت مهارکنندگی( MIC ) و حداقل غلظت کشندگی ( MBC ) عصاره‌ها استفاده گردید. تجزیه و تحلیل داده‌ها با استفاده از آزمون آنالیز واریانس انجام شد.   یافته‌ها: عصاره­های متانولی و استونی گال خرنوک اثرات ضدباکتریایی قابل قبولی علیه باکتری­های مورد آزمایش داشتند. بین فعالیت ضدباکتریایی عصاره­های متانولی و استونی گال و غلظت عصاره‌ها ارتباط معناداری وجود داشت(05/0> (p . اثر ضد باکتریایی عصاره­های متانولی و استونی گال خرنوک بر باکتری استافیلوکوکوس آرئوس بیشتر از باسیلوس سرئوس و باسیلوس سوبتیلیس بود. حداقل غلظت مهارکنندگی عصاره‌های گال خرنوک mg/ml 56/1 تا 13/3 بود. حداقل غلظت کشندگی عصاره‌های گال خرنوک mg/ml 13/3 تا25/6 بدست آمد.   مقدمه: گال بلوط گونه دارمازو به عنوان داروی گیاهی سابقه استفاده طولانی دارد. گال خرنوک در اثر رشد غیرطبیعی بافت گیاهی تحت تأثیر واکنش زنبورها بر روی جوانه‌های جانبی و انتهایی سرشاخه‌های درخت بلوط گونه دارمازو به وجود می‌آید. در این پژوهش خاصیت ضد باکتریایی عصاره‌های متانولی و استونی گال‌ Andricus moreae با نام‌ محلی خرنوک از درخت بلوط گونه Quercus infectoria بر باکتری‌های گرم مثبت شامل استافیلوکوکوس آرئوس، باسیلوس سرئوس و باسیلوس سوبتیلیس مورد بررسی قرار گرفت.

Title: اثرات تزریق محیطی عصاره بابونه بر علایم قطع مصرف مرفین در موش سفید صحرایی

http://yafte.lums.ac.ir/article-1-1270-fa.html

مقدمه:گیاه بابونه(Matricaria chamomilla) دارای فلاونوئید آپی ژنین می‌باشد که از خود اثرات شبه بنزودیازپینی نشان می‌دهد و بنابراین ممکن است در درمان ترک اعتیاد مفید باشد. در این تحقیق اثرات تزریق محیطی( درون صفاقی )عصاره هیدروالکلی بابونه بر علایم قطع مصرف مرفین در موش سفید صحرایی مورد بررسی قرار گرفت. بحث و نتیجه‌گیری: تزریق درون صفاقی عصاره بابونه می‌تواند علائم مهم سندرم ترک اعتیاد به مرفین را در موش صحرایی کاهش دهد. بنابر این لازم است اجزای موثر آن شناسایی و اثر آن در انسان نیز مورد بررسی قرار گیرد. یافته‌ها: نتایج نشان داد که تزریق درون صفاقی عصاره بابونه هرسه دوز تجویز شده، میزان بروزعلایم ترک مانند پریدن و بالارفتن را به صورت وابسته به دوز کاهش داد(001/0< P) مواد و روش‌ها: 24 موش نر (200تا250گرم) از نژاد ویستار انتخاب و با تزریق مرفین بصورت روزی دو بار به مدت 7 روز معتاد شدند. سپس موش ها به 4 گروه 6 تایی تقسیم شدند: گروه کنترل، که سالین و سه گروه بابونه، که عصاره بابونه(10،25و50 میلی گرم بر کیلوگرم)،30 دقیقه قبل از تزریق نالوکسان(5 میلی گرم بر کیلوگرم) به صورت درون صفاقی دریافت کردند.در تمام گروه ها 3ساعت بعد از آخرین تزریق مرفین، نالوکسان تزریق وعلائم ترک همچون پریدن و بالارفتن برای 30 دقیقه مطالعه شد. داده ها به دست آمده با انجام آزمون ANOVA مورد بررسی قرار گرفت.

Title: تأثیر گل‌مغربی و ویتاگنوس بر مستالژی دوره‌ای در مقایسه با ویتامین E: کارآزمایی بالینی تصادفی شده

http://yafte.lums.ac.ir/article-1-1271-fa.html

مقدمه: مستالژی یکی از شایع‌ترین دلایل نگرانی زنان است که باعث مراجعه مکرر آنها جهت مشاوره به مراکز مراقبت‌های بهداشتی می-شود. هدف کلی این پژوهش، مقایسه‌ی تأثیر گل مغربی و ویتاگنوس بر ماستالژی دوره‌ای بوده است. بحث و نتیجه‌گیری: ویتاگنوس، اثرات درمانی خوبی برای درمان مستالژی دوره‌ای دارد و می‌تواند به عنوان روش درمانی اثربخش مورد استفاده قرار گیرد. یافته‌ها: نتایج نشان داد که میانگین معیار درد در سه گروه بعد از مداخله کاهش یافته است و میانگین روزهای بدون درد در سه گروه افزایش داشته است و میانگین روزهای با درد متوسط در سه گروه کاهش یافته است و میانگین روزهای با درد شدید در سه گروه کاهش داشته است (چارت کاردیف) (0001/0< P). آزمون‌های آماری نشان داد که اثر ویتاگنوس بیشتر از ماده دیگر بوده است (0001/0< P). مواد و روش‌ها: این مطالعه یک کارآزمایی‌های بالینی یک مرحله‌ای است. نمونه پژوهش 210 زن مبتلا به مستالژی که در سه گروه هفتاد نفره قرار گرفتند. در گروه اول، قرص ویتاگنوس، گروه دوم کپسول گل‌مغربی و گروه شاهد ویتامین E به مدت دو ماه داده ‌شد. قبل و در دو ماه بعد از مداخله نمره درد سینه توسط پرسشنامه ارزیابی و با هم مقایسه شد. نتایج با استفاده از نرم افزار SPSS مورد تحلیل قرار گرفت. در این بررسی از آمار توصیفی و استنباطی شامل آزمون K.S، مان‌ویتنی‌یو، ANOVA و آزمون t زوجی استفاده شد.

Title: معرفی گیاه دارویی دارمازو (Quercus infectoria Oliv)در کوههای زاگرس و تعیین DNA بارکدینگ آن

http://yafte.lums.ac.ir/article-1-1272-fa.html

مقدمه: گیاهQuercus infectoria Oliv. یکی از مهمترین گونه‌های دارویی رشته کوه زاگرس می‌باشد که از زمان‌های قدیم به عنوان یک گیاه دارویی شناخته شده مورد توجه بوده است. مطالعات نشان داده درخت دارمازو دارای دامنه وسیعی از خواص دارویی می‌باشد. در این پژوهش معرفی جامع گیاه دارویی درخت دارمازو، محل های پراکنش و روش های صحیح تشخیص آنها ارائه گردیده است. بحث و نتیجه‌گیری: معرفی گیاه دارویی دارمازو در راستای شناسایی و بهره برداری صحیح و اصولی از این گونه با ارزش داروئی صورت گرفته و با تعیین DNA بارکدینگ آن، میزان خلوص و یا تقلبی بودن نمونه‌های موجود در بازار سنجیده می‌شود. یافته‌ها: بلوط دارمازو، حالت رسیدگی یکساله را نشان می دهند، در حالی‌که سایر بلوط‌ها حالت رسیدگی دو ساله را نشان می‌دهند. این گونه پیاله فاقد پایک بلند می‌باشد. معمولاً برگهای جوان دارای حاشیه صاف و برگهای مسن در حاشیه دندانه‌دار می باشند. تصاویر میکروسکوپی الکترونی نشان داد که برگها عمدتاً صاف و بدون کرک یا دارای کرکهای تنک و پراکنده هستند. توالی نوکلئوتیدها شامل حدود 680 جفت باز DNA می‌باشد. مواد و روش‌ها: نمونه‌ها از محل رویشگاه طبیعی در مناطق مختلف زاگرس جمع آوری گردیدند. بعد از شناسایی، برگهای سالم برای استخراج DNA و مطالعات میکروسکوپ الکترونی مورد استفاده قرار گرفتند. برگ نمونه‌های تازه برای حفظ کیفیت DNAبر روی سیلیکاژل خشک شدند. بعد از استخراج DNA، تخلیص محصول PCR و تعیین توالی، با استفاده از نرم افزار Sequencher توالی نوکلئوتیدها ویرایش شدند.

Title: اثر عصاره آبی گرده نخل بر نسبت جنسیت نوزادان در موش‌‌های سوری نژاد BALB/c

http://yafte.lums.ac.ir/article-1-1273-fa.html

مقدمه: فرزند سالم با جنسیت دلخواه یکی ازبزرگترین آرزوهای هر زوج می‌باشد. از بخش‌های مختلف خرما در طب سنتی برای درمان اختلالات تولیدمثلی استفاده می‌گردد. لذا این مطالعه با هدف بررسی اثر عصاره آبی گرده نخل بر نسبت جنسیت نوزادان در موش‌های سوری انجام گرفت. بحث و نتیجه‌گیری: احتمالاً اثر افزایش نوزادان نر نسبت به ماده با تجویز عصاره آبی گرده نخل را می‌توان به داشتن املاح معدنی غنی نظیر پتاسیم و سدیم نسبت داد که نیازمند تحقیقات بیوشیمیایی بیشتر در آینده می‌باشد. یافته‌ها: با توجه به یافته های ما در این مطالعه عصاره آبی گرده نخل باعث افزایش معنی‌دار نسبت نوزادان نر نسبت به نوزادان ماده می‌شود. مواد و روش‌ها: در این پژوهش از موش‌های کوچک آزمایشگاهی با وزن تقریبی 35-30 گرم در گروه‌های کنترل، شاهد و تجربی نر و ماده مورد مطالعه قرار گرفتند. گروه‌های تجربی نر و ماده خود به سه دسته 8 تایی تقسیم شدند که به ترتیب دوزهای mg/kg 100،200 و 400 عصاره آبی گرده نخل را در هر روز به مدت 35 روز دریافت کردند و در روز آخر تجویز موش‌های نر و ماده از گروه‌های مختلف با هم جفت گیری نموده و پس از تولد نوزادان نسبت جنسیت آنها مورد بررسی قرار گرفت. داده‌ها با کمک آزمون‌های ANOVA، Duncan وt Wilcox on Tes مورد ارزیابی قرار گرفت.
